# Brain‐oscillation-synchronized stimulation to enhance motor recovery in early subacute stroke: a randomized controlled double‐blind three‐ arm parallel‐group exploratory trial comparing personalized, non‐ personalized and sham repetitive transcranial magnetic stimulation (Acronym: BOSS-STROKE)

**DOI:** 10.1186/s12883-023-03235-1

**Published:** 2023-05-25

**Authors:** Anne Lieb, Brigitte Zrenner, Christoph Zrenner, Gábor Kozák, Peter Martus, Christian Grefkes, Ulf Ziemann

**Affiliations:** 1 Department of Neurology and Stroke, and Hertie-Institute for Clinical Brain Research, University of Tübingen, Tübingen, Germany; 2 Temerty Centre for Therapeutic Brain Intervention, Centre for Addiction and Mental Health, Toronto, ON Canada; 3 Department of Psychiatry, University of Toronto, Toronto, ON Canada; 4 Institute for Biomedical Engineering, University of Toronto, Toronto, ON Canada; 5 Institute for Clinical Epidemiology and Applied Statistics, University of Tübingen, Tübingen, Germany; 6 Department of Neurology, Goethe University Frankfurt, Frankfurt am Main, Germany

**Keywords:** Transcranial magnetic stimulation (TMS), Brain oscillation, Brain-state-dependent stimulation, Sensorimotor µ-oscillation, TMS-EEG, Personalized treatment

## Abstract

**Background:**

Stroke is a major cause of death and the most frequent cause of permanent disability in western countries. Repetitive transcranial brain stimulation (rTMS) has been used to enhance neuronal plasticity after stroke, yet with only moderate effect sizes. Here we will apply a highly innovative technology that synchronizes rTMS to specific brain states identified by real-time analysis of electroencephalography.

**Methods:**

One hundred forty-four patients with early subacute ischemic motor stroke will be included in a multicenter 3-arm parallel, randomized, double-blind, standard rTMS and sham rTMS-controlled exploratory trial in Germany. In the experimental condition, rTMS will be synchronized to the trough of the sensorimotor µ-oscillation, a high-excitability state, over ipsilesional motor cortex. In the standard rTMS control condition the identical protocol will be applied, but non-synchronized to the ongoing µ-oscillation. In the sham condition, the same µ-oscillation-synchronized protocol as in experimental condition will be applied, but with ineffective rTMS, using the sham side of an active/placebo TMS coil. The treatment will be performed over five consecutive work days (1,200 pulses per day, 6,000 pulses total). The primary endpoint will be motor performance after the last treatment session as measured by the Fugl-Meyer Assessment Upper Extremity.

**Discussion:**

This study investigates, for the first time, the therapeutic efficacy of personalized, brain-state-dependent rTMS. We hypothesize that synchronization of rTMS with a high-excitability state will lead to significantly stronger improvement of paretic upper extremity motor function than standard or sham rTMS. Positive results may catalyze a paradigm-shift towards personalized brain-state-dependent stimulation therapies.

**Trial registration:**

This study was registered at ClinicalTrials.gov (NCT05600374) on 10–21-2022.

## Introduction

### Background and rationale {6a}

Stroke is a major cause of death and the most frequent cause of permanent disability in western countries, with the absolute number of disease‐adjusted life years associated with stroke rising globally [1, 2]. The standard treatment of patients with debilitating deficits following stroke is task‐specific occupational therapy, e.g., physical therapy. However, many patients spend several weeks and months in rehabilitation clinics and years on conventional therapy modalities with often insufficient functional improvement. Despite improvements in rehabilitation programs, a significant number of patients remain with relevant disability with respect to functional independence and social participation. The success of motor restorative therapy is limited and mainly restricted to behavioral training [3]. The rising number of stroke survivors highly demands innovative therapies for brain restoration in patients suffering from disabling neurological deficits after stroke. Transcranial magnetic stimulation (TMS) therapy provides the possibility of inducing pro‐regenerative plastic changes not only in the stimulated brain tissue but also at the network level [4]. Those changes allow rehabilitation and conventional therapies to be much more effective. The dominant pathophysiological model underlying therapeutic approaches with non‐invasive brain stimulation in stroke is a disbalance in interhemispheric inhibition, where an increase in excitability of the (under‐active and over‐inhibited) ipsilesional hemisphere or a decrease in excitability of the (over‐active and under‐inhibited) contralesional hemisphere is achieved with regular high‐frequency repetitive TMS (rTMS) of the ipsilesional hemisphere or low‐frequency rTMS of the contralesional hemisphere [5, 6]. The neuromodulatory goal of these interventions is t increase cortical excitability of the ipsilesional hemisphere. In terms of therapeutic efficacy, conventional non‐synchronized rTMS has been categorized as “Level B: probable beneficial impact” based on 7 trials [7].

Recent findings indicate that the induction of a long‐term potentiation (LTP)‐like neuromodulatory effect is significantly increased when rTMS is synchronized with real‐time EEG‐defined high‐excitability states (specifically, the trough of the ongoing sensorimotor μ‐oscillation) as repeatedly demonstrated in our work on healthy participants [8–12] and patients with drug‐resistant major depressive disorder [13]. These findings are grounded in the neurophysiology of synaptic plasticity, where a coincidence between excitatory postsynaptic potentials (that can be measured in the EEG) with action potentials (evoked by TMS) underlies the induction of LTP, a principle that is referred to as spike‐timing dependent plasticity [14]. It has also been demonstrated that these oscillations can be accurately targeted in patients after stroke [15].

### Objectives {7}

The BOSS‐STROKE trial is based on the hypothesis that the timing of individual stimuli of the rTMS protocol is critically important for the therapeutic efficacy of rTMS and, specifically, that high‐frequency rTMS needs to be applied during the brief states of high corticospinal excitability to effectively support functional reorganization of brain networks in the early neurorehabilitation phase following stroke. Therefore, the trial will address a critical question: to what extent the strong evidence in basic neuroscience and human neurophysiological experimentation supporting the principle of spike‐timing‐dependent plasticity can be translated into a relevant clinical benefit for the treatment of stroke patients. All trial‐participating patients will receive standard treatment care according to national stroke therapy guidelines. The trial‐specific treatment will be applied additionally. All participating patients in this randomized controlled trial will receive the additional treatment, with one‐third receiving brain-state-synchronized TMS therapy (experimental condition), one‐third receiving non‐synchronized TMS therapy (control condition I) and one‐third receiving sham TMS therapy (control condition II). Regarding diagnostic procedures, all participating patients will be submitted to standard diagnostic procedures in stroke care. Trial‐specific will be the additional performance of a diagnostic EEG (to achieve a higher level of patient safety) and an MRI (to achieve a higher level of diagnostic and treatment precision), which are optional procedures in standard stroke care.

### Trial design {8}

The study is a multicenter randomized controlled double‐blind three‐arm parallel‐group exploratory clinical trial. Patients and raters in the post‐ and the follow‐up assessment will be blinded to the intervention condition the patient receives. A blinding of the medical doctor and/or study personnel conducting the intervention treatment is not possible, since setting‐up and controlling the intervention treatment requires knowledge of the intervention condition.

The study is being conducted as a Medical Device Regulation (MDR) clinical trial. The investigational device (bossdevice, sync2brain GmbH, Tübingen, Germany) is a real‐time digital signal processor that acts as a “brain oscillation state sensor” and consists of hardware and software algorithms. The technology was developed within our group, supported by a transfer of research grant (EXIST) by the German Federal Ministry for Economic Affairs. The device is now being commercialized by a spin-off company, sync2brain GmbH (Tübingen, Germany) under the name “bossdevice”. It is designed to read‐in a real‐time raw data stream from a bio‐signal amplifier (electroencephalography, EEG), to continuously analyze this data and to detect patterns based on oscillations in different frequencies. When such a specific bio‐signal pattern is detected, the device indicates this through a standard output port. This enables a connected device to determine with millisecond accuracy when a specific bio‐signal pattern occurs.

## Methods: participants, interventions, outcomes

### Study setting {9}

All experiments will be performed in a suitable experimental location with a qualified medical doctor according to the MDR prerequisites available on site at the University Hospital. Several national study centers will be participating in the trial. All study centers will be using identical equipment and carry out the study procedures under the same conditions. The list of participating study centers can be obtained from the coordinating investigator.

### Eligibility criteria {10}

#### Subject inclusion criteria

Subjects meeting all of the following criteria will be considered for admission to the trial:

- ▪ Age ≥ 18 years at the time of signing the informed consent.
- ▪ Cerebral ischemia identified by brain imaging (cerebral MRI or CT) occurred 1‐14 days ago.
- ▪ Subject understands and voluntarily signs an informed consent document prior to any study related assessments/procedures.
- ▪ Stroke has resulted in a new arm‐/hand motor deficit with ≤ 50 points in the FMA‐UE.
- ▪ Presence of motor evoked potentials (MEPs) in the paretic hand.
  - If no MEPs can be obtained in the resting muscle, MEP search during isometric contraction of pprox.. 10–20% maximum voluntary contraction (MVC) in the target muscle is to be applied.
  - If no MEPs can be obtained under contraction, MEP search with TMS double pulses (interstimulus interval (ISI) of 15 ms [16] is to be applied.
  - If still no MEPs can be obtained, TMS double pulses are to be applied during isometric contraction.
  - If no MEPs can be obtained, MEP search procedure can be repeated later up to 14 days after stroke onset
- **▪** The sensorimotor μ‐oscillation is recordable by EEG in the ipsilesional sensorimotor cortex as a power spectral density peak in the alpha frequency range (7-12 Hz) with a signal‐to‐noise ratio of at least 3 dB as compared to the aperiodic fractal background component of the spectrum.
- **▪** Subject is able to adhere to the study visit schedule and other protocol requirements.

#### Subject exclusion criteria:

Subjects presenting with any of the following criteria will not be included in the trial:

- **▪** Hemorrhagic stroke (this refers to primary intracerebral hemorrhage only; hemorrhagic transformation of ischemic infarcts is not an exclusion criterion)
- **▪** Estimated life expectancy < 12 months
- **▪** Presence of intracranial ferromagnetic metal (extracranial stents ≥10 cm away from the TMS coil are acceptable) in accordance with current safety guidelines [17]
- **▪** Intraocular metal, cochlear implants
- **▪** If TMS might interact with sensors of active implants (e.g., intra‐cardiac defibrillators).
- **▪** If a cranial bone gap affects currents induced by TMS in the brain (such as after craniotomy).
- **▪** History of seizures or epilepsy.
- **▪** Treatment intervention can’t be started within 14 days after onset of stroke.
- **▪** Women during pregnancy and lactation.
- **▪** Participation in other clinical trials or observation period of competing trials.
- **▪** Persistent addiction disorder (except for nicotine dependence)
- **▪** CNS malignoma
- **▪** If there is any concern by the investigator regarding the safe participation of the subject in the study or for any other reason the investigator considers the subject inappropriate for participation in the study.
- **▪** The ability to consent in patients who are unable to speak will be assessed on the basis of the NIHSS by an independent physician.

The presence of a cardiac pacemaker is no longer an exclusion criterion, as TMS can be safely operated if at least 20 cm away from the pacemaker [17]. Prior ischemic events are not an exclusion criterion as this would unduly limit the patient population.

### Trial center requirements

The requirements for the participation of a study center in the study are, on the one hand, defined by the legal regulations for the conduct of clinical studies reviewed by the responsible ethics committee of the coordinating center. With regard to study-specific requirements for a study center’s participation in the study, all sites must have long standing expertise with TMS in stroke trials and authority to fully access patients on their Stroke Units for trial recruitment.

The following components are required at the participating centers to perform the intervention:

- **▪** TMS Stimulator R30 or X100 (MagVenture, Denmark), with EEG filter and coil holder
- **▪** TMS Active/Placebo coil Cool-B70 A/P (MagVenture, Denmark)
- **▪** TMS Coil Cooling System (MagVenture, Denmark)
- **▪** EEG/EMG Amplifier actiCHamp Plus 32 (BrainProducts GmbH, Germany)
- **▪** TMS-compatible EEG Caps, sizes 54, 56 and 58 cm (EasyCap GmbH, Germany)
- **▪** Neuronavigation System, e.g., Localite GmbH, Germany
- **▪** Real-time digital signal processing device and control software (bossdevice, sync2brain GmbH, Tübingen, Germany)
- **▪** Windows Control PC
- **▪** Treatment chair and vacuum pillow to position the patient’s head

Funding for equipment not available at some of the study centers was obtained as part of the funding application. The equipment is provided to the centers free of charge via a loan agreement. The real-time signal processing device (bossdevice) is provided free of charge by sync2brain GmbH.

### Who will take informed consent? {26a}

Patients will be checked for eligibility by a study physician after admission due to acute ischemic stroke. Study inclusion is possible from the day of admission. Each eligible patient will be informed about the modalities of the clinical investigation in accordance with the provided patient informed consent (IC). The patient is to be informed both in writing and verbally by the investigator before any study‐specific procedure is performed. The patient will be given sufficient time (i.e., > 24 h) to decide whether to participate in this study and to ask questions concerning this trial. The patient must give consent in writing. The patient and informing physician must each personally date and sign the informed consent form with an integrated declaration on data privacy protection.

### Patients

The target population of this study comprises acute stroke patients with predominantly motor deficits of the upper extremity, who are able to understand and assess the dimension of the study adequately in order to provide informed consent.

In case the study participant is unable to write legibly, but can understand the patient information, the following procedure will be followed: If the study participant is able to speak, oral instead of written consent will be given in the presence of at least one independent witness who was also involved in informing the study participant. The consent given orally will be documented in writing, dated, and signed by the witness. In case the study participant is unable to articulate, the capacity to consent is additionally checked and confirmed by an independent physician. An independent physician will determine the ability of the study participant to give informed consent, based on the following reproducible parameters. These parameters correspond to the score from listed National Institute of Health Stroke Score (NIHSS) items (1. level of consciousness, 2. orientation, 3. following commands, 4. language/aphasia). If the study participant is unable to speak and scores > 1 in at least one subscale of those NIHSS items, the consent cannot be given and the patient cannot participate in the study. Patients with comprehension difficulties who are mentally unable to fully comprehend the consent process are also not allowed to participate in the study.

### Additional consent provisions for collection and use of participant data and biological specimens {26b}

An additional consent form for data collection with a detailed description of the data collection method, information about the patient’s rights and contact information in case of questions is attached to the trial consent form. Biological samples will not be collected.

## Interventions

### Intervention description {11a}

High‐frequency rTMS will be applied to the ipsilesional motor cortex in 400 bursts of 100 Hz triplets with a mean inter‐burst interval of 3 s (20 min treatment duration, 1,200 pulses per day) for five consecutive workdays (6,000 pulses total), followed by 40 min task‐specific hand/arm‐physiotherapy. The physiotherapy will be applied at all study centers according to a uniform standard operating procedure, which is based on standard treatment procedures in stroke care. Stimulation will be provided in a dedicated room by a GCP‐qualified trial physician or trial technician under supervision of a medical doctor. For the primary endpoint, FMA-UE raters will be trained to improve inter-rater reliability.

This trial has one Experimental Condition and the Control Conditions 1 and 2. The three conditions will be randomized 1:1:1. All conditions target the hand representation of the ipsilesional motor cortex.

### Experimental condition (personalized stimulation)

Each 100 Hz triplet is triggered by the real-time digital signal processor, when it detects a EEG‐defined state of high corticospinal excitability (the trough of the ongoing sensorimotor μ‐oscillation). The stimulation intensity will be set to 80% of the resting (or active, see below) motor threshold in the first dorsal interosseus muscle of the paretic hand. A mean interburst interval of 3 s will be targeted by adjusting the power threshold of the μ‐oscillation in the EEG, leading to 20 min treatment duration.

### Control condition 1 (non-personalized stimulation)

The identical rTMS protocol as in Experimental Condition, but 100 Hz triplets will not be synchronized to the ongoing sensorimotor μ‐oscillation.

### Control condition 2 (sham stimulation)

The same protocol as in the Experimental Condition synchronized to the EEG‐defined high excitability state (trough of the ongoing µ-rhythm), but with ineffective rTMS, using the sham side of an active/placebo TMS coil designed for double‐blind clinical trials.

### Explanation for the choice of comparators {6b}

The comparison between the Experimental Condition and Control Condition 1 will reveal to what extent brain‐oscillation‐synchronized rTMS is more effective than non‐brain oscillation‐synchronized but otherwise identical rTMS, i.e., it will address the comparison of primary interest in this trial. The comparison between the Experimental Condition and Control Condition 2 will address the important question to what extent it is the real brain stimulation rather than somatosensory and auditory inputs synchronized to the ongoing brain oscillation that causes the therapeutic effect. Use of a combined active/passive stimulation coil with realistic auditory and somatosensory stimulation enables stimulation that blinds both the patient and the treatment provider to the condition.

The dose (number of stimuli per session, number of sessions) was adopted from previous stroke trials that used high‐frequency rTMS of the ipsilesional motor cortex in early subacute stroke patients [18–21].

### Study visit procedures

#### Screening visit

Screening (V1) will be performed within maximal 14 days prior to first treatment (V2). After having signed the informed consent, patients will undergo all assessments listed below:

- Physical and neurological examination and medical history. This includes documentation of the NIHSS, stroke lesion size and site, and major medical complications (e.g. pneumonia).
- Clinical Assessments and Tests:
  - ◦ FMA‐UE; assessment at screening should be repeated within 3 days prior first treatment (V2), if the screening visit was done earlier
  - ◦ Relative grip strength measurement (vigorimeter) of the affected compared to the non‐affected hand. The best of 3 attempts will be documented; assessment at screening should be repeated within 3 days prior first treatment (V2), if the screening visit was done earlier.
  - ◦ Stroke‐specific Quality‐of‐Life Scale (SS‐QOL)
  - ◦ Modified Rankin Scale Score
  - ◦ Barthel Index

- EEG measurement (to be repeated within 3 days prior first treatment (V2), if the screening visit was done earlier):
-
  - ◦ Preparation of 32‐channel EEG. Recording of a 5 min resting‐state eyes‐open EEG and subsequent analysis of the signal‐to‐noise (SNR) ratio of the peak of the µ‐oscillation (frequency band 7‐12 Hz) over the ipsilesional sensorimotor area in the C3 (stroke in the left hemisphere) or C4 (stroke in the right hemisphere)‐Hjorth montage.
  - ◦ Exclusion of subjects with an insufficient SNR of the sensorimotor µ‐rhythm below 3 dB
  - ◦ If applicable a diagnostic EEG measurement can be obtained to screen for abnormal EEG activity (e.g., interictal epileptic discharges) prior to treatment (not relevant for inclusion, sufficient data of one standard EEG can also be obtained from an EEG before screening).
- TMS measurement (to be repeated within 3 days prior first treatment (V2), if the screening visit was done earlier):
  - ◦ Determination of the TMS hotspot over the hand representation of the ipsilesional motor cortex for eliciting MEPs of maximum amplitude in the first dorsal interosseus muscle of the paretic hand [please cite [22]].
  - ◦ Extended MEP search procedure (only in case if no MEPs can be obtained with standard single-pulse TMS in the resting muscle): MEP search during isometric tonic contraction of 10–20% maximum voluntary contraction (MVC) of the first dorsal interosseus muscle of the paretic hand.
  - ◦ If no MEPs can be obtained during contraction, MEP search with TMS double pulses (interstimulus interval (ISI) of 15 ms [16]) will be applied in the resting target muscle.
  - ◦ If still no MEPs can be obtained, TMS double pulses will be applied during isometric contraction.
  - ◦ If no MEPs can be obtained, the MEP search procedure can be repeated later up to 14 days after stroke onset.
  - ◦ If still no MEPs can be obtained after 14 days, the patient will be excluded.
  - ◦ Otherwise, the resting or active motor threshold in the first dorsal interosseus muscle of the paretic hand will be determined with the method that elicited MEPs. The motor threshold is defined in the resting muscle as the lowest TMS stimulator intensity that results in small MEPs > 50 µV peak-to-peak amplitude in > 5/10 trials. In the active muscle it is the lowest TMS stimulator intensity that results in MEPs > 200 µV in the average of 5 trials [22]. Motor threshold will be expressed as percentage of maximum stimulator output (%MSO).
- Neuroimaging session procedures for measurement of anatomical MRI
  - ◦ In case the patient has already received a diagnostic MRI scan between onset of stroke and participation in the study, an additional anatomical MRI is required only if the diagnostic MRI scan in not suitable for the navigation system to guide TMS.
  - ◦ If the patient has not yet received an MRI between the onset of stroke and participation in the study or the existing MRI is not sufficient, an anatomical MRI (MPRAGE sequence, TE = 2.18 ms, TR = 2300 ms, TI = 1100 ms, flip angle = 9°, 192 slices, voxel size = 1 × 1 × 1 mm, DWI, FLAIR) should be obtained.
  - ◦ In the case of MRI contraindications TMS will be applied at the location of the identified hand motor hotspot. Neuronavigation (Localite GmbH) based on MNI‐brain adaption will then be employed to maintain a consistent coil position, orientation and angulation throughout the sessions of a given patient using skull and facial landmarks rather than the anatomical MRI data.
- Randomization and Stratification: At the end of the screening visit and after checking all inclusion and exclusion criteria, the eligible study participants will be assigned to their respective treatment by randomization.
  - ◦ 1:1:1 randomization by biostatistical center
  - ◦ stratification will be applied with regard to:
    - **▪** study center
    - **▪** motor threshold (<60 %; >60 % MSO)

The following procedures will be performed on 5 consecutive working days (V2-V6):

- Assessment of possible adverse events from previous session
- Preparation of 5‐channel EEG
- Determination of TMS hotspot over hand representation of ipsilesional motor cortex
- Determination of resting (or active) motor threshold
- rTMS intervention (personalized rTMS or non‐personalized rTMS or sham rTMS), 400 bursts of 100 Hz triplets (i.e., 1,200 pulses per session), interburst interval ∼3 s, stimulus intensity 80 % resting (or active) motor threshold.
- Documentation of session time, parameters and assessment of adverse events
- Physiotherapy session (40 min) immediately after rTMS intervention

#### Post-treatment clinical assessment

Performed by a blinded rater according to the pre‐treatment clinical assessment visit on the same day of the last treatment (V7):

- FMA‐UE
- Relative grip strength measurement (vigorimetry)
- Stroke‐specific Quality‐of‐Life Scale (SS‐QOL)
- Modified Rankin Scale Score
- Barthel Index

#### Follow-up visit

The last visit will be scheduled 3 months ± 7 days after the last treatment and will be performed by a blinded rater according to the pre‐treatment clinical assessment visit:

- FMA‐UE
- Relative grip strength measurement (vigorimetry)
- Stroke‐specific Quality‐of‐Life Scale (SS‐QOL)
- Modified Rankin Scale Score
- Barthel Index
- Number of days as an inpatient during the 3 months after the intervention
- Number of days in inpatient rehabilitation

### Criteria for discontinuing or modifying allocated interventions {11b}

In the event that the stimulation is not tolerable for the patient, it will be discussed with the patient whether they would like to retry at a later time (within the specified 14 days after the stroke event, s. eligibility criteria) or whether the study participation will be terminated. Modifications of the intervention are not planned to be performed.

Reasons for premature termination of an individual trial subject are:

- Death
- Withdrawal of consent
- Patient lost to follow‐up
- Major protocol violation
- Occurrence of an Adverse Event or a Severe Adverse Event (e.g., seizures) that prevent the patient’s further safe participation in the trail.
- If, in the investigator’s opinion, continuation of the trial would be detrimental to the subject’s well‐being.
- pregnancy
- Noncompliance

The PI decides on the withdrawal of a patient from study treatment in case one of the criteria mentioned above occurs. The reason for withdrawal will be recorded in the CRF as well as in the subject’s medical records. Premature termination should be prevented as best as possible. In case a subject withdraws from further participation at their own request, the reasons/circumstances will be determined and documented. All sessions and assessments of the last trial day will be performed and documented as well as the final status of the patient. Each withdrawn patient should enter the follow-up phase. The patient will not suffer any disadvantage in case of requested withdrawal of any further study procedure participation including the follow up.. Adverse Events (AEs)/ Serious Adverse Events (SAEs) should be followed‐up as far as possible.. Patients will be contacted by phone or letter prior to follow‐up examinations. Subjects dropping out after completion of intervention therapy will not be replaced. A final assessment visit for the relevant safety data will be always offered to the patient.

### Stopping rules and exclusion of study centers

Participating centers will be discontinued if their recruitment rate is less than 50% of the agreed recruitment rate indicated in the declaration of commitment at mid-recruitment (15 months into the recruitment period), and either no efforts have been undertaken to improve recruitment rate, or all opportunities for improving recruitment rate have been exhausted. The Data Safety and Monitoring Board (DSMB) has the right to discontinue the trial prematurely, in case of at least one of the following situations: Severe adverse events, substantial changes in risk–benefit considerations, insufficient recruitment.

### Strategies to improve adherence to interventions {11c}

The compliance is expected to be very high as the rTMS treatment is painless and non-invasive, and communicative barriers such as severe aphasia are excluded. Accordingly, in one high-frequency rTMS trial with comparable design only 4/157 (2.5%) subacute stroke patients dropped out because they did not tolerate the treatment [21].

### Relevant concomitant care permitted or prohibited during the trial {11d}

During the study, all patients will receive standard conventional rehabilitation treatment including occupational therapy after TMS treatment for 40 min, 5 times per week. The treatment includes task‐oriented training that involves active participation of the affected limb and individualized motor task training. Subsequently, exercise training, including active‐assistive range of motion exercise of the affected extremity, holding, moving, releasing of cups and cubes, will be administered by the same therapists.

According to the exclusion criteria participation in competing trials or therapeutic interventions other than conventional rehabilitation treatment procedures with effect on motor recovery, will have to result in termination of study participation. Patients are informed about the regulations before giving informed consent.

### Provisions for post-trial care {30}

There will be no special procedures following for patients that have completed the trail. Patients will be treated according to the standard of care after the termination of the study. After the last treatment, the subject enters the Follow‐up phase. For patients prematurely terminating the study for one of the above listed reasons, there will be no special follow‐up procedures. In case of a premature termination of therapy, reasons/circumstances and if applicable the final status will be documented. If the patient does not withdraw the consent for further Follow‐up, he/she should be followed‐up as planned. To those who suffer harm from trial participation (e.g., epileptic seizure, headache), the standard treatment for the symptoms that have been arisen will be provided in the hospital.

### Outcomes {12}

#### Primary clinical endpoint

Primary efficacy endpoint is the motor performance after the intervention, as assessed by the Fugl‐Meyer assessment of the upper extremity (FMA‐UE). The upper‐extremity (UE) portion of the Fugl‐Meyer assessment (FMA‐UE, range 0‐66, 0 = no motor function, 66 = normal motor function) is the most frequently used scale to quantify post‐stroke motor recovery of the upper extremity [23] and correlates well with subjective assessment of motor function. The FMA‐UE was used as an endpoint in most of the recent high‐frequency rTMS trials in early subacute stroke patients [18–21]. For these reasons, the FMA‐UE immediately after the last treatment session was chosen as the primary outcome measure of this trial.

#### Secondary endpoints

1. Motor performance 3 months after the intervention, as assessed by the FMA‐UE [24].
2. Relative grip strength measured with a vigorimeter. Grip strength is an additional robust measure that can be quantified easily by vigorimetry (measured in kg). Relative grip strength is defined as of the maximum grip strength of the affected hand divided by the maximum grip strength of the unaffected hand. It is unaffected by compensatory movement strategies, and has been successfully used in stroke trials previously [25].
3. Stroke‐Specific Quality‐of‐Life Scale (SS‐QOL). The SS‐QOL is a patient‐centered outcome measure designed to provide an assessment of health‐related quality‐of life specific to patients with stroke [26, 27].
4. Modified Rankin Scale (mRS) score. The modified Rankin Scale (range 0‐6, 0 = no disability, 6 = death) is the most widely used scale for measuring the degree of disability or dependence in the daily activities of people who have suffered a stroke [28].
5. Barthel Index (BI). The Barthel index (ordinal scale 0‐100, 0 = fully dependent, 100 = independent in feeding, walking and grooming) [29] is an index of independence in activities of daily living and is used to monitor functional outcome during neurorehabilitation in stroke [30].
6. Number of days as an inpatient during the 3 months after the intervention.
7. Number of days in inpatient rehabilitation.

All the above endpoints have been previously validated and are listed in the relevant stroke treatment guidelines [24–30]. Assessments will be performed before, immediately after, and 3 months after the intervention to capture short‐ and long‐term effects by a rater blinded to treatment condition. Additionally, the number of days as an inpatient during the 3 months after the intervention is considered as a socioeconomically relevant measure of how quickly a patient can return to his/her home environment.

### Participant timeline {13}

The study is planned to span a total duration of 42 months, between November 2022 and May 2026. The TMS intervention duration for each subject is 1 week (5 sessions per week). The maximum study duration including screening, MRI scan, TMS intervention and a 3-month follow-up measurement for each subject will be 3.5 months. The time course of events can be seen in Table 1.

**Table 1:** Flow chart of visits and events

**Events**	**Screening (V1)**-14 days to -1 day prior 1th treatment	**Treatment (V2-6)** (treatment will be performed on 5 following working days)	**Post –treatment (V7)**^**g**^ (last treatment and post- treatment visit will be performed on the same day)	**Follow-up (V8)**^**g**^(after 3 months ± 7 days)
Study Entry				
Informed Consent (IC)	X	–	–	–
Inclusion/Exclusion Criteria	X	–	–	–
Physical and neurological examination	X	–	–	–
Medical History	X	–	–	–
Pre-treatment clinical assessment^a^	X^f^	–	–	–
TMS and EEG measurement^b^	X^f^	–	–	–
MRI^c^	X	–	–	–
Randomization^d^	X	–	–	–
TMS and physiotherapy sessions	–	X	–	–
Post-treatment assessment^a^	–	–	X	–
Post-treatment follow-up assessment ^e^	–	–	–	X
Adverse Events	–	Start of study treatment or after first treatment and until 7 days after discontinuation from treatment		

^a^pre & post treatment assessment and tests: Fugl‐Meyer Assessment upper extremity (FMA-UE); grip strength; SS‐QOL; Rankin Scale; Barthel Index. For screening, FMA-UE and grip strength should be repeated within 3 days prior first treatment (V2), if the screening was done earlier

^b^Each center will send the EEG raw (32‐channel) data to the coordinating center. It is sufficient that appropriate data from a standard EEG measurement is available. This can also be from an examination before screening

^c^MRI is not mandatory for inclusion, but a sufficient imaging should be available before start of treatment (please

see section “screening visit V1” for more details)

^d^in case of eligibility the randomization will take place at the end of the screening

^e^post treatment follow-up assessment and tests: FMA-UE; grip strength; SS‐QOL; modified Rankin Scale; Barthel Index; number of days as an inpatient during the 3 months after the intervention

^f^ TMS, EEG (32‐channel) and Pre‐treatment clinical assessment at screening should be repeated within 3 days prior first treatment (V2), if done earlier. Standard EEG is not relevant for inclusion and can be performed at any time, also before screening

^g^Performed by a blinded rater

### Sample size {14}

It is planned to recruit 144 patients for the study. This corresponds to a recruitment rate of approximately 10 patients per study site and year. The primary objective is to establish whether personalized stimulation (Experimental Condition) is superior to non-personalized stimulation (Control Condition 1) and sham stimulation (Control Condition 2) regarding differences in FMA-UE at the end of intervention. A mean FMA-UE at baseline of 33 ± 12 [31] is expected. From the guideline [24] a minimal clinically significant improvement of 12 points on FMA-UE is derived, which leads to an effect size of 1.0. However, an effect size of 0.67 instead of 1.0 will be conservatively assumed, as a large placebo effect in the sham condition might be observed, and also in the non-personalized stimulation condition. The analysis will be done as an analysis of covariance (baseline adjusted) with overall F-test for the factor “study arm”. If the F-test is significant, according to the closed testing procedure (Hochberg Tamhane, Multiple Comparison Procedures, New York, 1987) no adjustment for multiple testing will be necessary for pairwise comparisons in the three arm situation. Assuming normal distribution of the primary outcome measure and an R-squared of 0.2 for the covariate (baseline FMA-UE) 36 evaluable patients per arm will be needed and, thus, 108 evaluable patients in total for the F-test (type 1 error 0.05, power 80%). This is based on the worst case scenario of an effect size for non-personalized stimulation = 0.67/2 = 0.335. Thus, on the one hand, it is ensured that in the case of unexpectedly positive results of the non-personalized stimulation, there will be enough power to proceed to subsequent pairwise comparisons. On the other hand, if standardized differences between study arms of 0.67 hold, there will be a power of 88% to confirm these differences (type 1 error 0.05, two-sided). To adjust for degrees of freedom for center, baseline, and 20% dropouts, 144 patients will be included, i.e., 48 per study arm. The sample size calculation was performed by PASS 2020.

### Recruitment {15}

The six participating sites treat a total of approx. 6,000 acute stroke patients per year (~ 15,000 during the 30-months recruitment period), of which 144 patients will be included (i.e., ~ 1% of the stroke population handled at the study sites, and on average 10–11 patients per study site per year). Similar recruitment rates have been achieved in the targeted patient group previously by the applicants in pilot studies in Tübingen and Cologne [32]. All sites have long standing expertise with TMS in stroke trials and authority to fully access patients on their Stroke Units for trial recruitment. According to the Declarations of Commitment of the study sites, there is confidence that recruitment will be feasible as planned.

The following overall dropout rates (i.e., loss to follow-up for all kinds of reasons) were reported in the high-frequency rTMS trials in early subacute stroke patients with similar trial designs: 17% of 157 included patients [21], 20% of 69 patients [18], 5% of 41 patients [19], 24% of 42 patients [20], 17% of 24 patients [2]. On average, the dropout rate in these trials was 17.1%.

## Methods: assignment of interventions

### Sequence generation {16a}

Patients will be randomized to one of the three study conditions/arms (1:1:1) and will be stratified according to study center and motor threshold (≤ 60%; > 60%MSO). The biostatistical center produced a randomization list (block randomization) with varying block length and stratified for study center to realize a 1:1:1 randomization. The randomization of each participant will be conducted centrally by the biostatistical centre via electronic interaction. The operator at each center will be responsible to implement the randomized treatment set-up. Patients and raters will be blind to the rTMS condition.

### Implementation {16c}

Patients will be recruited in the participating study centers after admission due to acute ischemic stroke. Recruiting is possible from the day of admission. Patients fulfilling all inclusion and none of the exclusion criteria will be enrolled into the study. Randomization will be performed electronically via the database by any study team member other than the rater at the end of the screening visit. The randomization sequence will then be generated by the biostatistical center and will be sent to the designated operator via email.

### Concealment mechanism {16b}

The sequence consists of numbers and one letter. The number is entered in the stimulator and determines whether the active or placebo side of the symmetrical coil will be used. The letter is entered in the control software of the real-time digital signal processor and determines whether pulses are administered synchronized or non-synchronized to EEG.

## Assignment of interventions: blinding

### Who will be blinded {17a}

The patient as well as the rater will be blinded to the intervention condition the patient receives. A blinding of the medical doctor and/or study personnel conducting the intervention treatment is not possible, since setting‐up as well as controlling the intervention treatment requires knowledge of the intervention condition.

### Procedure for unblinding if needed {17b}

A randomized log is kept at the study center by the study personnel conducting the intervention treatment at the study center. This list contains patient name, patient study ID and the assigned treatment condition. The study personnel has to ensure that the log is kept at a safe place, that is accessible in case of emergency unblinding. The operator and study physicians will have access to the un-blinding list, if necessary for medical reasons. The actual allocation must not be disclosed to the patient and/or other study personnel including other site personnel, monitors, corporate sponsors or project office staff. Investigators are encouraged to discuss with the principal investigators if they believe that unblinding is necessary. Premature unblinding of study participant has to be considered in case of severe adverse events or medical emergency. The reason for unblinding, date and time, and names of the study team members will be documented on the un-blinding log. The monitor and the biostatistical center will be informed.

## Data collection and management

### Plans for assessment and collection of outcomes {18a}

Patient assessment source data will be managed following Good Clinical Practice (GCP) guidelines using an FDA-conforming database with an audit trail and internal monitoring provided by the coordinating center. Participating centers will submit data using electronic case report forms (eCRF). The name, day of the month, and month of birth of participants will not be stored electronically, participant data records will be identified with identifier unique to each participant. Corresponding personal data will be maintained in paper format by each participating center and with a copy (via fax) of forms containing the participant’s name and contact data (such as consent forms) transmitted to the coordinating center. All paper data will be maintained in a locked room and locked cabinet. The informed consent process is documented in the patient records. The original signed documents will be part of the investigator’s site file and retained with it and one copy included the insurance policy of the trial will be handed to the patient. All procedures administered to the subjects on entry to the trial or at any time during the trial will be documented paper-based in a pseudonymous manner. At the end of each visit, the data will be entered into The Clinical Data Management System [“secuTrial”], which will be used for data capture, processing and storage of study data. The paper-based documentation will be stored in a lockable cabinet at the investigational site, not-accessible to non-authorized personnel. Data entry is performed access-controlled at the investigational site by clinical staff after having received training and a user manual for the electronic CRF. Raw biosignal data will be uploaded to a secure cloud server at the coordinating center for quality control and analysis. Various EEG and EMG measures (spectral analysis, signal-to-noise ratio, motor evoked response amplitudes, etc.) will be extracted by the coordinating center and stored in the trial database.

### Plans to promote participant retention and complete follow-up {18b}

There is existing evidence that early subacute patients receiving real rTMS are likely to have a therapeutic benefit on their neurorehabilitation [7]. Patients in the Experimental Condition (personalized stimulation) will receive real rTMS stimulation. In addition, the stimulation will be personalized to their individual brain rhythm, for which the study hypothesizes a further increase in beneficial effects. Patients in Control Condition 1 (non‐personalized stimulation) will receive real rTMS. As mentioned above, there is existing evidence for a beneficial effect of real rTMS in this patient population. Patients in Control Condition 2 (sham stimulation) are not expected to benefit from the sham stimulation, however, they receive task‐specific hand‐arm physiotherapy every day until the end of the therapy visits. The beneficial rTMS effects remain to be demonstrated, however, patients have the chance to receive additional treatment beyond standard treatment that can promote the motor recovery of their paretic arm while keeping the risk of side effects particularly low. Therefore, it is expected that there will be a high level of interest and compliance on the part of patients. In terms of the follow-up visit 3 months post-intervention we offer reimbursement for travel costs including a travel‐accident insurance. Each study participant is insured against any health impairment occurring as a result of participation in the study in accordance with the applicable laws and regulations.

### Data management {19}

The trial Case Report Form (CRF) is the primary data collection instrument for the trial. For this project, electronic Case Report Forms (eCRFs) will be used. The Clinical Data Management System [“SecuTrial”] is validated and changes are tracked via an audit trail. The Center for Clinical Trials Tübingen (ZKS) will monitor the correct completion / perform plausibility checks of the data stored in the eCRFs to avoid discrepancies with the source data. Implausible or missing data will be queried. The data from all centers will be entered in a central data base (secuTrial) provided by the Institute for Clinical Epidemiology and applied Biostatistics (IKEaB, University Tübingen). The system secuTrial is validated and used by several centers for clinical trials in Germany. It is linked to the central data center of the University Hospital Tübingen (UKT) and to which only authorized staff of the IKEaB has access. The data managing plan (including digital data storing and archiving) of the IKEaB will comply with all legal requirements. The system secuTrial provides an audit trail for all activities (data entry, modification, and deletion). A role system allows access to selected patients and different rights (reading, writing) for different investigators. The IKEaB will provide an intensive training for all users of this system during the study.

The correctness of entries in CRFs will be confirmed by dated signature of an authorized investigator or delegated investigator. Data will be entered into the eCRF using an access-controlled, GCP compliant, validated and audit-trailed system by authorized staff of the investigational site. Plausibility of the data will be checked as implemented in the CRF. Entered data will be monitored on a regular basis.. Implausible data and missing data will be queried. The database will be locked after completion of entry, cleaning of data and final data review. All relevant trial data and documents (CRF-worksheets, source data and Investigator Site File (ISF) including subject identification list and relevant correspondence) will be archived by the principle investigators for 10 years according to local law or regulations.

### Confidentiality {27}

All data will be handled in accordance to the European General Data Protection Regulation and the applicable local data protection regulations as well as the applicable regulations for the clinical trial. Subjects will be informed about data safety in the trial and have to give written consent to collect and process their data as well as to the transfer of their data in a pseudonymous way. The privacy policy has been reviewed by the responsible ethics committee. Data capture records, trial reports and communications and drug accountability records identifies the patient by an assigned identity number to maintain privacy. It must be determined by the principle investigators who is authorized to view personal data. The Patient Identification Log can be accessed only by the authorized study personnel. To prevent unauthorized access (electronically and physically) restricted access rights to pseudonymized data are implemented. All data will be stored either paper-based or electronically in a pseudonymous manner and handled strictly confidential. Data will be processed at the investigation site in accordance to the safety concept of the institution. Extensive back‐up procedures are implemented to strictly avoid loss of data. All legal requirements that concern data protection and data confidentiality will be respected thoroughly. Every authorized person is sworn to secrecy. Data of withdrawn patients will be stored and further used subjected to the patient’s consent. Data that is no longer used will be deleted immediately.. After the end of the archiving period, all data will be deleted according to data protection law.

### Plans for collection, laboratory evaluation and storage of biological specimens for genetic or molecular analysis in this trial/future use {33}

Not applicable, no samples collected.

## Statistical methods

### Methods and analyses {20a, 20b, 20c, 21b}

The primary population is the intent‐to‐treat population. This population is defined as all randomized patients with Baseline measurement of the primary endpoint (inclusion criterium). The primary analysis will be done as Baseline‐adjusted analysis of covariance, adjusted for study center with a type 1 error of 0.05 (two‐sided for pairwise comparisons) with primary endpoint FMA‐UE measured at the last therapy visit. The study arm will be coded as a three level factor and in the first step an F‐Test for the overall null hypothesis (all three means are equal) vs. the alternative (at least two measures are different) will be done. If this test is significant, all three pairwise comparisons will be performed without correction for multiple testing. If the F‐Test is not significant, p‐values for pairwise comparisons will be calculated but have to be interpreted descriptively. In the case of a significant result (for the F‐Test) in this step, following the principle of testing hierarchically ordered hypotheses, the same strategy will be applied for follow‐up after three months. For the parameters of interest (effects within study arms and their differences between study arms) two‐sided 95% confidence limits will be calculated. In case of relevant imbalances between study arms regarding known prognostic factors, sensitivity analyses will be done adjusted for these factors. Analogous methods will be applied for the analysis of secondary endpoints (proportional odds model for ordinally scaled variables, logistic regression for binary variables, if possible baseline adjusted). Even though p‐values will be reported, all results have to be interpreted descriptively. Descriptive analyses will include means, standard deviations, and ranges for continuous normally distributed data. Medians and interquartile ranges will be calculated for non‐normally distributed data. Nominal, especially binary variables will be described using absolute and relative frequencies. Additionally, we will present line listings for AEs/SAEs. Missing values of primary and secondary endpoints will be imputed multiply using the method of Rubin. Predictor variables will be age (years), gender, study center and Baseline, which will be available in the intent‐to‐treat population (inclusion criterion). We will use 500 imputation samples; the seed will be the calendarial date of the first programming of the analysis program. An interims analysis is not planned. Exploratory subgroup analyses will be done for gender. Before breaking the code of the randomization a statistical analysis plan will be produced. Deviations from this plan will be documented and justified.. In case of premature termination of study centers or severe imbalance of prognostic factors between study centers, a sensitivity analysis non‐stratified for study centers will be presented.

### Plans to give access to the full protocol, participant level-data and statistical code {31c}

The study protocol will be available on https://www.medizin.uni-tuebingen.de/de/das-klinikum/einrichtungen/kliniken/neurologie/studien. Participant-level data can be provided anonymously.

All investigators have access to the final data set. Data acquired will be stored for at least 10 years in accordance with current data protection law.

## Oversight and monitoring

### Composition of the coordinating centre and trial steering committee {5d}

Sponsor of the trial is the University Hospital of Tübingen with Prof. Ulf Ziemann, Director of the Department of Neurology & Stroke, as Sponsor Deputy and Principle Investigator. Coordinating Investigator is Dr. Sven Poli, Deputy Director of the Department of Neurology & Stroke. Biometrician is Prof. Peter Martus, Director of the Institute for clinical epidemiology and applied biometry, who will also provide and supervise the data management. The Project Management is carried out by the Centre for Clinical Studies (ZKS) of the University Hospital of Tübingen that will also provide monitoring, SAE-management and -reporting. An independent external international scientific advisory board is associated that will provide scientific guidance, and will also take responsibility as the Data Safety and Monitoring Board (DSMB). Manufacturer of the investigational device (bossdevice) is sync2brain GmbH that will provide technical support. At the coordinating center the study takes place in the DIN-ISO-certified TMS study center of the University Hospital Tübingen with an on-site study team with high expertise in performing TMS in stroke patients, certified study assistants with expertise in study-associated clinical ratings of stroke patients and medical doctors with research focus in TMS and neurorehabilitation of stroke patients. The Brain Network and Plasticity research group at the Hertie Institute for Clinical Brain Research with world-leading expertise in TMS, led by Prof. Ziemann, supports the trial in data analyses.

### Composition of the data monitoring committee {21a}

An independent external international scientific advisory board will be associated with the BOSS-STROKE trial that will provide scientific guidance, and will also take responsibility as the Data Safety and Monitoring Board (DSMB). Six researchers with international renowned expertise in stroke trials and/or rTMS treatment and an independent biometrician have agreed to participate in the board. The DSMB will be informed of all safety-related events by the PI. The DSMB will meet in regular intervals every 12 months and monitor the safety and adequate course of the clinical trial. It has also to decide whether reasons for premature termination of the clinical trial have occurred (see also above, criteria for discontinuing or modifying allocated interventions). Based on its review the DSMB will provide recommendations regarding trial modification, continuation or termination. The DSMB members will have access to the unblinded data if deemed necessary for their evaluations. The DSMB Charter is available for review upon request at the coordinating study center.

### Adverse event reporting and harms {22}

Adverse events will be collected, reported and assessed using standardized procedures in accordance with the regulatory requirements. The management and reporting of adverse events is supervised by the Pharmacovigilance Department at the Clinical Trials Center (ZKS Tübingen). For the purpose of this trial, the period of observation for collection of adverse events extends from the first use of the device until 7 days after the last application of the device. Any deterioration in the state of consciousness or neurological, especially motor, deficits will be recorded during the study. The assessment of the severity of the event is in accordance to the Common Terminology Criteria for Adverse Events (CTCAE) criteria (version 5.0). The relationship between the use of the medical device and the occurrence of each adverse event will be assessed for causality and categorized by the sponsor investigator and the second assessor.

#### Harms

Repetitive transcranial magnetic stimulation (rTMS) is considered a non‐invasive, painless and safe method to treat patients with neurological or psychiatric disorders as long as the safety guidelines of the International Federation of Clinical Neurophysiology [17] are followed. There is worldwide experience with hundreds of thousands of patients treated with rTMS, and a high class of evidence for therapeutic efficacy has been demonstrated for several indications [7]. A very rare adverse event is an epileptic seizure induced by rTMS. Strokes imply an increased risk of epileptic seizures, but there is no evidence that rTMS is associated with a higher risk of seizure initiation in stroke patients compared with subjects without brain damage [33]. In the proposed BOSS‐STROKE study, this risk is likely even further minimized compared to other studies, because patients are monitored by continuous EEG and, thus, early epileptic seizure detection is possible and rTMS treatment can be stopped immediately.

EEG and electromyography (EMG) passively record electrical biosignals and have no side effects; irritation to the skin may be provoked by application of the electrode cream (EEG) or adhesive electrodes (EMG).

MRI is a very commonly applied diagnostic method. Severe adverse effects of magnetic fields and radio frequency are not known, as long as the standardized MRI inclusion / exclusion criteria are obeyed. Possible side effects are muscle twitching or irritation of peripheral nerves while the patient is inside of the MRI-scanner. Other side effects are headaches and tinnitus as well as heating of metallic tattoos.

All experiments will be performed in a suitable location in a University Hospital with a qualified medical doctor available on site throughout the treatment sessions.

#### Expected adverse events (AE), frequency and relief or treatment (see Table 2***)***

**Table 2:** Expected adverse events (AE), frequency and relief or treatment

AE	Likelihood of occurrence	Relief/ Treatment	Prevention
Seizure	Very low	rTMS halt, treatment according to neurological emergency standards	Monitoring by EEG during rTMS
Slight/moderate Headache	High	usually subside spontaneously, standard analgesics (NSAIDs)	Not applicable
Hearing impaired	Very low	rTMS halt, treatment according to otolaryngology emergency standards	patients and treatment providers will be protected by earplugs
Syncope	Low	rTMS halt, treatment according to neurological emergency standards	Monitoring by EEG during rTMS
Presyncope	Low	rTMS halted, treatment according to neurological emergency standards	Monitoring by EEG during rTMS
slight/moderate (Local) Pain	High	usually subside spontaneously, standard analgesics (NSAIDs)	Not applicable
Dizziness	Low	rTMS halt	Not applicable

Expected adverse events are:

- slight headache
- slight discomfort on the skin surface
- mild transient discomfort at the site of stimulation during stimulation

#### Adverse events that do not have to be reported

More than half of patients experience apparent post-rTMS adverse events, but a substantial proportion of these reported events are rather not related to the investigational treatment but occur as a consequence of the experienced stroke. This may lead to lower specificity for genuine complications of novel treatments and falsely inflate the number of adverse events associated with the therapy. Therefore, events that can be excluded from expedited reporting will be specified (Table 3a-b):

**Table 3:** Adverse events associated with stroke, that do not have to be reported

a) Acute stroke-related deficits	
New neurological deficits which may appear within several days after stroke	Abnormal gaze (partial gaze palsy, forced deviation)
	Visual field (partial hemianopia, complete hemianopia, bilateral hemianopia)
	Facial paresis (minor paresis, partial paresis, complete palsy)
	Limb ataxia
	Sensory deficits (pinpricks)
	Dysarthria (mild to moderate, unintelligible)
	Neglect (partial, complete)
	Speech/comprehension deficits (mild to moderate aphasia, severe aphasia, mute)
Neurological deficits which may display post-stroke	Deterioration in level of consciousness
	Deterioration of neurologic deficits, especially motor deficits
Complications due to stroke	Cerebral edema, cerebral incarceration
	Need for external ventricular drainage or decompression surgery
b) Other Adverse Events (AEs)	
AEs that are likely observed in stroke patients	• falls with subsequent fractures • subsequent stroke or transitory ischemic attack • heart infarction • pneumonia • urinary tract infection • sepsis • dehydration • delirium

Patients with stroke may present with a variety of neurologic symptoms including alterations in vision, changes in speech, focal numbness or weakness, disequilibrium or alteration in level of consciousness, dystextia, dystypia, dysgraphia, aphasia, apraxia, neglect, as well as elementary motor, ataxic, and sensory deficits. Some new deficits may occur within the early subacute period after stroke, or already present ones can deteriorate poststroke (Table 3a). Other complications are likely observed in stroke patients in the early subacute period but do not qualify as adverse events with a relation to the rTMS treatment and therefore will not be reported (Table 3b).

### Frequency and plans for auditing trial conduct {23}

Monitoring for this study is provided by the Clinical Trial Center Tübingen (ZKS Tübingen). The monitoring will be conducted according to ZKS Tübingen internal Standard Operating Procedures (SOPs) and a dedicated monitoring manual for the study. The monitoring timelines include, for all centers, pre‐study visit, initiation visit, regular monitoring visits during the course of the trial as well as a close out visit. Usually, monitoring will end with the last visit after full documentation of the last patient enrolled (close out visit). All investigators agree that the monitors regularly visit the trial site, assure that the monitors will receive appropriate support in their activities and will have access to all trial‐related documents. In addition to the monitoring activities, audits can be conducted by the sponsor or assigned auditors. These audits may include checking the whole course of the study, documentation, trial center, investigators and the monitor. The competent regulatory authorities may also conduct inspections. With their participation in the study, the investigator agrees to support the activities of the auditor/inspector, provide them with direct access to the source documents, study documentation and give them the opportunity to audit/inspect the study site, laboratory facilities, storage of the investigational product, etc.

### Plans for communicating important protocol amendments to relevant parties (e.g. trial participants, ethical committees) {25}

Communication of important protocol amendments to relevant parties will be managed by the project management of the trial at the Clinical Trial Center Tübingen in consultation with the principle investigator. The communication with trial participants is handled by the study team via consultation at the study visits or other communication channels preferred by the participant.

### Dissemination plans {31a}

The results of this study will be disseminated via open-access publications in peer-reviewed journals, and presentations in local, national and international meetings and conferences, exclusively using anonymized data. All disseminations related to this study are the responsibility of the principal coordinating investigator and the authorship will reflect the contributions of each collaborating center. Any publication, abstract or presentation based on patients included in this study will be approved by the principal coordinating investigator in line with the current recommendations of the International Committee of Medical Journal Editors (http://www.icmje.org/ Recommendations for the Conduct, Reporting, Editing and Publication of Scholarly Work in Medical Journals 01/10/2021 Available from: 2019). The reporting timelines are based on the requirements of the Medical Device Coordination Group (MDCG) guideline 2020‐10/1 "Safety reporting in clinical investigations of medical devices under the Regulation (EU) 2017/745".

## Discussion

The BOSS-STROKE trial is an exploratory phase-3 trial to provide positive evidence for the main hypothesis, i.e., brain-oscillation-synchronized rTMS is more effective than standard non-brain-oscillation-synchronized rTMS and sham rTMS in improving arm-/hand function of patients with early subacute stroke. We hypothesize that synchronization of rTMS with the phase of the ongoing sensorimotor oscillation indicating high corticospinal excitability leads to significantly stronger improvement of paretic upper limb motor function than the same rTMS protocol non‐synchronized to the ongoing sensorimotor oscillation or sham stimulation. The objective of this clinical trial is to investigate the therapeutic efficacy of rTMS synchronization to targeted EEG‐states in the early subacute phase after ischemic stroke to improve upper limb motor rehabilitation.

The risk to patients participating in this trial is low and not exceeding the expected benefit: Early subacute stroke patients receiving real rTMS are likely to have a therapeutic benefit on their neurorehabilitation [7]. Patients in Experimental Condition (personalized stimulation) will receive real rTMS stimulation personalized to their individual brain rhythm, for which the study hypothesizes an extra increase in beneficial effects [9]. However, this effect remains to be demonstrated by the study. Patients in Control Condition 1 (non‐personalized stimulation) will receive real rTMS non-synchronized to the ongoing sensorimotor rhythm. There is evidence for a beneficial effect of real rTMS in this patient population [7]. Patients in Control Condition 2 (sham stimulation) are not expected to benefit from the sham stimulation; however, they have minimal discomfort by the treatment sessions and no risk of adverse effects from sham rTMS. All patients receive task‐specific hand‐arm physiotherapy, for which a definite beneficial effect for motor recovery after stroke has been demonstrated [3].

In case of the confirmation of the main hypothesis for the primary endpoint of this trial, i.e., brain-oscillation-synchronized rTMS results in a higher FMA-UE immediately after the last treatment session compared to the two control conditions, non-brain-oscillation-synchronized rTMS and sham rTMS, a confirmatory trial will be conducted. The confirmatory trial will seek to provide firm evidence for efficacy and safety of brain-oscillation synchronized rTMS in an even broader range of early subacute stroke patients with a broader variety of arm-/hand dysfunction severity. The anticipated clinical impact of the confirmatory trial is to provide firm evidence for superiority of brain-oscillation-synchronized personalized therapeutic brain stimulation with respect to clinically relevant outcome of arm-/hand function in early subacute stroke patients. If successful this will lead to a significant expansion of the treatment repertoire of this patient population in the early neurorehabilitation setting.

In summary, the results of this trial will be of outstanding importance for the establishment of novel personalized brain‐oscillation synchronized rTMS therapy protocols, which likely will lead to a paradigm shift in non-invasive brain stimulation. This new therapy option will lead to a significant expansion of the recovery repertoire of patients suffering from a stroke in the early neurorehabilitation setting that might be expanded to a variety of other deficits (e.g., aphasia or neglect) and other brain network disorders in further confirmatory trials.

## Trial status

The coordinating study center has been initiated and started recruiting in December 2022. The participating study sites are in the initiation process and will start with recruitment within the next weeks. The recruitment period is planned to span a duration of 32 months.

## Data Availability

The datasets used and/or analysed during the current study are available from the corresponding author on reasonable request. Data acquired will be stored for at least 10 years in accordance with current data protection law.

## Appendix

- 1. Approval of the Ethics Committee dated 2022–04-06.
- 2. *English Translation* of the Approval of the Ethics Committee.
- 3. Approval of the Federal Institute for Drugs and Medical Devices dated 2022–09-12.
- 4. *English Translation* of the Approval of the Federal Institute for Drugs and Medical Devices.
- 5. Notification of grant by the Federal Ministry of Education and Research dated 2022–05-02.
- 6. *English Translation* of the notification of grant by the Federal Ministry of Education and Research.

## Abbreviations

AE: Adverse Event
BfArM: Federal Institute for medicinal products and medical devices
BMBF: Bundesministerium für Bildung und Forschung
CRF: Case Report Form
CTCAE: Common Terminology Criteria for Adverse Events
CI: Coordinating Investigator
CT: Computer Tomography
DSMB: Data and Safety Monitoring Board
eCRF: Electronic Case Report Form
EEG: Electroencephalography
EMG: Electromyography
FDA: U.S. Food and Drug Administration
FMA‐UE: Fugl ‐Meyer‐Assessment Upper Extremity
GCP: Good Clinical Practice
GDPR: General Data Protection Regulation
IC: Informed Consent
IFCN: International Federation of Clinical Neurophysiology
IKEAB: Institute for Clinical Epidemiology and Applied Statistics
ISF: Investigator Site File
ISI: Interstimulus Interval
ITT: Intention to Treat
LTP: Long term potentiation
MDCG: Medical Device Coordination Group
MDR: Medical Device Regulation
MEP: Motor Evoked Potential
MPDG: Medizinprodukterecht‐Durchführungsgesetz
MRI: Magnetic Resonance Imaging
MSO: Maximum Stimulator Output
MVC: Maximum voluntary contraction
NSAID: Non-steroidal anti-inflammatory drug
PI: Principal Investigator
RMT: Resting motor threshold
rTMS: Repetitive transcranial magnetic stimulation
SAE: Serious Adverse Event
SNR: Signal‐to‐noise ratio
SOP: Standard operating procedure
SS-QOL: Stroke Specific Quality Of Life Scale
TMS: Transcranial magnetic stimulation
UKT: University Hospital of Tübingen
ZKS: Clinical Trial Center (Zentrum für Klinische Studien)
